# Diploid‐ and tetraploid wall barley exhibit alternative molecular responses but comparable reproductive performance in response to different ambient temperature regimes

**DOI:** 10.1111/tpj.71051

**Published:** 2026-07-25

**Authors:** Timo Hellwig, Michelle Marianne Tonnelier, Helene Villhauer, Anna Bucharova, Maria von Korff

**Affiliations:** ^1^ Institute of Plant Genetics Heinrich‐Heine‐University Düsseldorf Düsseldorf Germany; ^2^ Conservation Biology Philipps University Marburg Marburg Germany; ^3^ Cluster of Excellence on Plant Sciences “From Complex Traits towards Synthetic Modules” Düsseldorf Germany

**Keywords:** allopolyploidy, heat stress, gene diversity, *Hordeum murinum*, homeolog expression bias, phenotypic plasticity, subgenome divergence, subgenome partitioning, transcriptomics

## Abstract

Diploid and allopolyploid relatives often differ in their responses to stressful or variable environments, but it remains unclear whether this is reflected in greater reproductive stability in such conditions, and whether any such advantage is associated with greater canalisation or plasticity of morphological and developmental traits or a more extensive transcriptional response. We tested this in the annual grass *Hordeum murinum* by comparing the reproductive performance, phenotypic responses, and transcriptomic responses of diploid and allotetraploid *H. murinum* subspecies under control and elevated temperature conditions. Gene‐level nucleotide diversity was higher in the diploid ssp. *glaucum* than in either subgenome of the allotetraploid ssp. *murinum*, whereas divergence was greatest between the two subgenomes of the allotetraploid. Relative to ssp. *glaucum*, ssp. *murinum* altered expression in a larger proportion of its analysed transcriptome (10% versus 8%), spanned more enriched functional categories (36 versus 19), and showed pronounced subgenome‐specific contributions to the response. In addition, 702 of 11 106 homeolog pairs showed temperature‐dependent shifts in expression bias despite the absence of genome‐wide dominance of either subgenome. By contrast, phenotypic responses were largely similar between subspecies. Although ssp. *glaucum* and ssp. *murinum* differed in phenotype, especially in phenology, temperature explained more phenotypic variation than subspecies and reduced reproductive biomass by 42% in ssp. *glaucum* and 47% in ssp. *murinum*. Overall, ssp. *murinum* showed neither greater plasticity nor better maintenance of reproductive performance. Thus, compared with diploid *H. murinum*, allopolyploid *H. murinum* showed broader transcriptional deployment under elevated temperature, but not greater reproductive stability or clear differences in the plasticity of morphological and developmental traits. These results point to a decoupling of trait and molecular differentiation from fitness under elevated temperature, suggesting that the allotetraploid's altered transcriptional response may represent an alternative route to maintaining fitness comparable to that of the diploid rather than a reproductive advantage.

## Significance Statement

Polyploid plants often possess broader genomic and regulatory repertoires that could alter their responses to environmental stress, but whether this translates into reproductive advantages remains unclear. In wall barley, elevated temperature triggered a broader and subgenome‐partitioned transcriptional response in the allotetraploid, but this did not result in greater phenotypic plasticity or better or more stable reproductive performance, showing that molecular flexibility, phenotypic response, and stress performance can be decoupled.

## INTRODUCTION

Polyploidy, the presence of more than two complete chromosome sets, has been a recurrent feature of plant evolution and is recognised as a major contributor to genome evolution and diversification in angiosperms (Heslop‐Harrison et al., 2023; Soltis et al., 2014, 2015; Soltis & Soltis, 2009; Wood et al., 2009). Whole‐genome duplication does not simply increase chromosome number. It can alter gene dosage, patterns of gene retention, and regulatory interactions, which reshape the genetic architecture on which evolution acts (Hu et al., 2021; Van de Peer et al., 2017, 2021).

This question becomes especially interesting in allopolyploids, which combine divergent parental genomes within a single nucleus. In such systems, the important issue is not only redundancy, but the coexistence of subgenomes with different evolutionary histories and regulatory backgrounds (An et al., 2024; Grover et al., 2012). As a result, the relative contribution of the two subgenomes can differ among genes, tissues, or environments, and expression bias may be gene‐specific and condition‐dependent rather than reflecting a fixed genome‐wide dominance pattern (Akama et al., 2014; Akhunova et al., 2010). Allopolyploids therefore provide a useful framework for asking not only how much variation is present, but also how that variation is transcriptionally deployed under environmental change.

These genomic properties have often been invoked to explain why polyploids are frequently associated with stressful, fluctuating, or recently disturbed environments (Mousavizadeh et al., 2022; Rice et al., 2019; Van de Peer et al., 2017). As a result, polyploids are often expected to fare better under stressful or fluctuating environments, and many polyploid species do appear to benefit from genome duplication under at least some conditions (Tossi et al., 2022; Zhang et al., 2020). However, a broader molecular repertoire does not necessarily lead to stronger phenotypic plasticity, greater stress tolerance, or improved short‐term performance. Therefore, recent reviews emphasise that the effects of polyploidy in changing environments on the organismal level are mixed across taxa and stressors, and that broad generalisations about a universal polyploid advantage remain difficult to sustain (Li et al., 2024; Soltis et al., 2014; Tossi et al., 2022). It is therefore useful to distinguish between different levels at which polyploidy may matter: how variation is arranged in the genome, how it is mobilised at the transcriptomic level, and how strongly those molecular differences are reflected in the phenotype.

Temperature and precipitation are two of the principal climatic axes structuring plant distributions and productivity across ecosystems (Harrison et al., 2020; Yao et al., 2022). Among environmental factors, elevated temperature provides a particularly informative test case because it can affect plant development, growth, and reproduction. In temperate cereals, warmer conditions are known to alter growth, flowering time, inflorescence development and yield‐related traits, even when temperatures remain below those causing acute heat damage (Jacott & Boden, 2020; Mishra et al., 2023). Key reproductive processes in wheat and barley are optimised, depending on developmental stage, at temperatures of approximately 10–20°C, and even modest warming of a few degrees above this range reduces growth, affects reproductive development and reduces spikelet and floret numbers, grain set, and ultimately yield, well before temperatures reach levels associated with acute heat damage (Ejaz & von Korff, 2017; Farooq et al., 2011; Jacott & Boden, 2020; Lan et al., 2025; Porter & Gawith, 1999). Elevated temperature, therefore, offers a useful framework for testing whether polyploidy affects molecular and phenotypic plasticity and how this translates into reproductive performance.

*Hordeum murinum* L., commonly known as wall or mouse barley, is a winter annual grass closely related to cultivated barley. It provides a useful system for examining how elevated temperature responses differ between diploid and polyploid lineages. *Hordeum murinum* is taxonomically complex, forming the *Hordeum murinum* aggregate, which includes three subspecies differing in ploidy: diploid ssp. *glaucum* (Steud.) Tzvelev (2n = 2x = 14), the tetraploid ssp. *murinum* L. (2n = 4x = 28), and the hexaploid ssp. *leporinum* (Link) Arcang. (Cuadrado et al., 2013). In this study, we focus on the diploid *H. murinum* ssp. *glaucum* and the allotetraploid *H. murinum* ssp. *murinum*. For simplicity, we refer to these subspecies as *glaucum* and *murinum* throughout the manuscript. Allotetraploid *murinum* originated approximately 454 thousand years ago through hybridisation between diploid *glaucum* and a second, now‐extinct diploid lineage that diverged from *glaucum* approximately 5 million years ago (Hellwig et al., 2025). Herein, we will follow the subgenome nomenclature of Rajhathy and Morrison (1962), who designated G for the *glaucum*‐derived subgenome and M for the subgenome from the extinct species. The subspecies also differ in ecology: diploid *glaucum* occurs mainly around the Mediterranean and into south‐central Asia, whereas tetraploid *murinum* occupies much of that range and extends further into temperate Central and Eastern Europe (Bieniek, 2018; Villhauer et al., 2026; von Bothmer et al., 1995). Such expanded distribution and broader ecological niches in allotetraploids compared with their diploid relatives are often observed and have been attributed to the fact that allotetraploids combine two subgenomes that may have evolved different adaptations before hybridisation (Parisod & Broennimann, 2016; Ramsey, 2011). This combination of related diploid and allotetraploid lineages, contrasting distributions, and identifiable subgenomes makes *H. murinum* well suited to investigate how polyploidy affects trait expression, phenotypic plasticity, and responses to environmental change in a widespread and ecologically versatile grass.

Here, we combine controlled‐environment phenotyping with population genomic and transcriptomic analyses to assess how the two cytotypes of *H. murinum* respond to elevated ambient temperature. Specifically, we ask: (i) how gene‐level diversity and divergence differ between diploid *glaucum* and the two subgenomes of allotetraploid *murinum*; (ii) whether elevated temperature induces distinct transcriptional responses in the allotetraploid, including subgenome‐specific expression patterns and shifts in homeolog expression bias; and (iii) whether diploid *glaucum* and allotetraploid *murinum* differ in phenotype, trait covariation, or plasticity under elevated temperature.

## RESULTS AND DISCUSSION

### Patterns of gene diversity and divergence in *glaucum* and *murinum*

**Figure 1:**
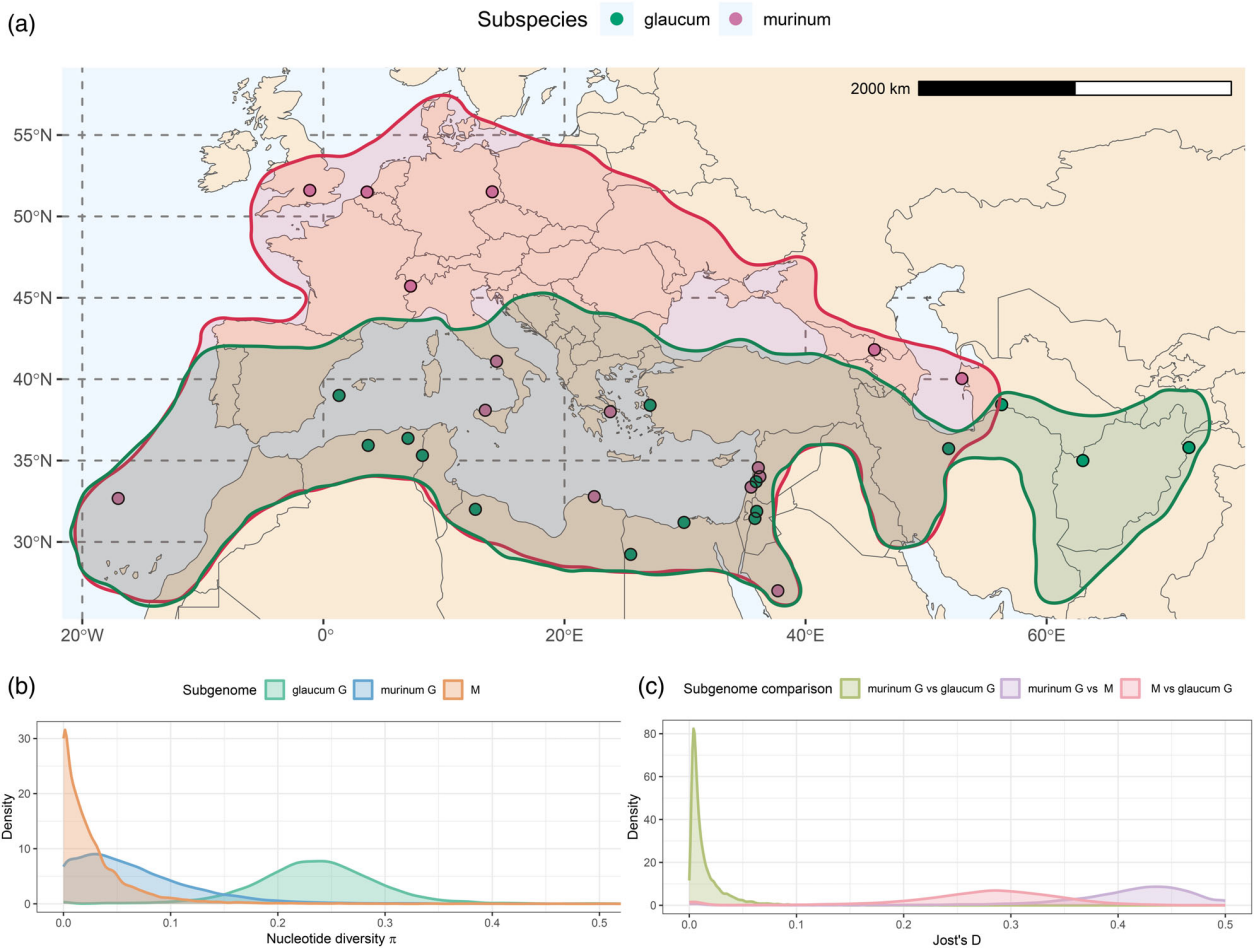
Geographic origins of utilised plant accessions sorted by subspecies. Shaded areas indicate the subspecies' distribution ranges (a). For metadata on the utilised accessions see Table S1. Density distributions of genes' nucleotide diversity (π; b) and genetic differentiation (Jost's D; c) across and between subgenomes.

To place the analyses of responses to elevated temperature in a genomic context, we first asked how genetic diversity is distributed in relation to ploidy in the accession panels used here, both between diploid *glaucum* and allotetraploid *murinum*, and between the two subgenomes of *murinum*. We compared diploid *glaucum* and allotetraploid *murinum* using 15 accessions per subspecies, selected from a larger collection to represent the geographic and climatic space occupied by each taxon (Villhauer et al., 2026; Figure 1a). Because tetraploid *murinum* combines a glaucum‐derived G subgenome with a second M subgenome, we separated *murinum* reads by subgenome and quantified gene‐level nucleotide diversity (π) within *glaucum* G, *murinum* G, and *murinum* M, as well as genetic divergence (Jost's D) between them. This provides the genomic backdrop for the transcriptomic and phenotypic comparisons that follow.

Average genetic diversity was lowest in the *murinum* M genome (π = 0.028), followed by *murinum* G (π = 0.066), which retained only 28% of the diversity observed in *glaucum* G (π = 0.236; Figure 1b, Table S2). This reduction may reflect the genetic bottlenecks that species often experience following polyploidisation (Baumel et al., 2002; Guo et al., 2013), and the recent demographic expansion observed in *murinum* may have further accentuated this pattern (unpublished).

Divergence was lowest between the two G genomes, intermediate between M and *glaucum* G, and highest between the M and *murinum* G subgenomes, indicating that the two *murinum* subgenomes are more strongly differentiated from one another than either is from diploid *glaucum* G. This pattern is consistent with continued divergence of the two subgenomes following polyploidisation and may be associated with subfunctionalisation or relaxed purifying selection (Flagel & Wendel, 2009). Thus, in the material analysed here, the diploid panel harbours more within‐genome sequence diversity, while the allotetraploid stores variation primarily in the form of divergence between its two subgenomes. This pattern is consistent with observations from other allopolyploids and suggests that the allotetraploid is not simply a redundant combination of its parental genomes, but a system in which polyploidisation may generate novelty through continued subgenome divergence and subsequent non‐additive evolutionary change (Buggs et al., 2011).

### Broader transcriptomic responses to elevated temperature in allotetraploid *murinum*

To characterise transcriptomic responses to elevated temperature in the material analysed here, we sequenced RNA from the second leaf collected 10 days after transfer to control and HT conditions and analysed differential expression separately within each subspecies.

Different temperature regimes induced differential expression in both subspecies, but the response was broader in *murinum*. In *glaucum*, we identified 1740 differentially expressed genes (DEGs), representing 7.94% of the analysed transcriptome; 1037 (4.73%) were upregulated and 703 (3.21%) were downregulated (Figure S1, Table S3). In *murinum*, 3769 genes were differentially expressed, corresponding to 10.20% of the analysed transcriptome, of which 2594 (7.02%) were upregulated and 1175 (3.18%) were downregulated (Fiugre S2, Table S4). The higher absolute number of DEGs in *murinum* is expected given its larger gene content, but the higher proportion of responsive genes is more informative here (odds ratio = 0.76; Fisher's Exact Test, *P* < 2.2e‐16).

**Figure 2:**
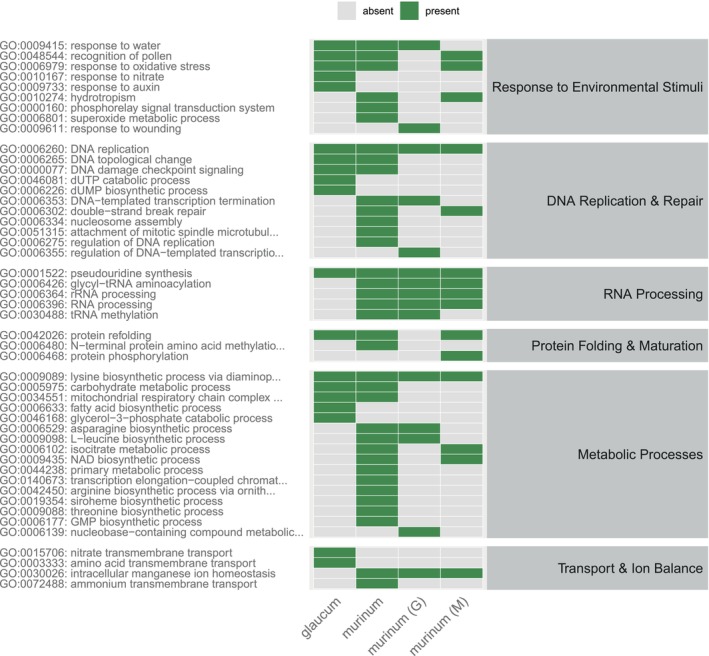
Significantly enriched GO terms in response to high ambient temperature separated by subspecies and subgenomes within allotetraploid *murinum*.

Several enriched GO terms were shared between the two subspecies (Figure 2; Table S5; Figures S3 and S4). In both *glaucum* and *murinum*, DEGs were enriched for ‘response to oxidative stress’ (GO:0006979) and ‘DNA replication’ (GO:0006260), and both also showed enrichment for ‘DNA damage checkpoint signalling’ (GO:0000077) and ‘response to water’ (GO:0009415). The induction of oxidative stress‐related genes (15 in *glaucum*, 20 in *murinum*; Tables S3 and S4) is consistent with a typical heat stress response, as elevated temperature results in increased membrane fluidity which can cause electron leakage and thereby promote the formation of reactive oxygen species (ROS; Hasanuzzaman et al., 2013). In line with this heat stress response, we also observed differential expression of typical heat stress‐related genes like heat shock proteins and heat stress transcription factors (10 in *glaucum*, 45 in *murinum*; Tables S3 and S4). The concurrent enrichment of ‘DNA damage checkpoint signalling’ (GO:0000077) and ‘DNA replication’ (GO:0006260) suggests that the stress induced by elevated temperature was sufficient to trigger genome‐maintenance responses, including checkpoint activation and DNA repair‐associated processes (Bita & Gerats, 2013; Nisa et al., 2019). These shared enrichments therefore point to a common heat stress core programme in both subspecies.

Beyond this shared core response, the two subspecies differed in the functional composition of their temperature‐responsive transcriptomes. This difference was apparent not only in the different number of DEGs but also in the breadth of functional enrichment: *glaucum* showed 19 significantly enriched GO terms, whereas *murinum* showed 36 (Figure 2; Table S5), indicating that elevated temperature affected a wider range of functional categories in the allotetraploid. In *glaucum*, uniquely enriched GO terms included ‘response to auxin’ (GO:0009733), transmembrane transport processes for nitrate and amino acids, and lipid‐related categories such as ‘fatty acid biosynthetic process’ (GO:0006633) and ‘glycerol‐3‐phosphate catabolism’ (GO:0046168). These functions point to hormonal adjustment, shifts in nutrient transport, and membrane remodelling, all of which are commonly associated with abiotic stress responses and the maintenance of cellular integrity under elevated temperature (Guerra et al., 2015; Liu et al., 2023; Shintani et al., 2024). In *murinum*, uniquely enriched terms included ‘RNA processing’ (GO:0006396), ‘rRNA processing’ (GO:0006364), ‘tRNA methylation’ (GO:0030488), and several biosynthetic processes, including amino acid and NAD biosynthesis (Figure 2). These categories suggest stronger post‐transcriptional regulation and broader metabolic reorganisation under elevated temperature (Coate et al., 2016; Lan et al., 2025; Muyle et al., 2022), which in an allotetraploid may reflect the added regulatory demands of coordinating two subgenomes under stress (Gu et al., 2014; Ling et al., 2021). Together, these results indicate that while both subspecies activate a partially shared transcriptional response, *glaucum* responds through a smaller set of adjustments, whereas *murinum* mobilises a more functionally diverse and regulatorily more complex transcriptomic response.

This broader response in *murinum* was also partitioned between its two subgenomes. Of the 3769 DEGs identified in *murinum*, 3538 could be assigned to 2604 orthogroups containing between one and six genes. Among these orthogroups, 766 were represented in both subgenomes, whereas 881 were unique to the G subgenome and 957 to the M subgenome. Several GO terms were likewise enriched in only one subgenome. Most notably, ‘response to oxidative stress’ (GO:0006979) was enriched only in the M subgenome, even though individual oxidative stress‐related genes, including several heme peroxidases, were also upregulated in G. The response in *murinum* was therefore not only broader than in *glaucum*; it also involved substantial subgenome‐specific contributions, with many orthogroups represented in only one subgenome and several GO terms enriched in only one of the two subgenomes. Such subgenome‐specific stress responsiveness has been reported in other allopolyploids (Lee & Adams, 2020; Liu et al., 2015) and is consistent with divergent subgenome functions, which may have resulted from divergence after polyploidisation or from differences retained from the ancestral genomes.

Taken together, elevated temperature altered gene expression in both subspecies, but allotetraploid *murinum* mobilised not only more DEGs in absolute terms, but also a larger proportion of its analysed transcriptome, and these genes spanned a broader range of functional categories than in diploid *glaucum*. The enriched functions were only partly overlapping, indicating that both subspecies activated a shared core transcriptional response, but differed in how broadly and through which additional functional pathways that response was deployed. The main difference in expression response between the two subspecies therefore lies in the extent and functional emphasis of that response, with allotetraploid *murinum* exhibiting a stronger transcriptional response than diploid *glaucum*.

### Environment‐dependent homeolog expression bias in allotetraploid *murinum*

**Figure 3:**
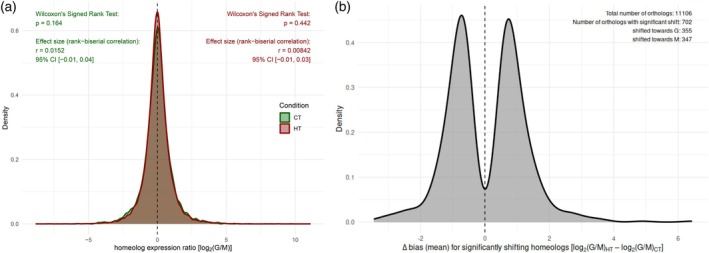
Homeolog expression bias and its association with divergence. Subgenome expression ratios denote of all genes in control (CT) and high‐temperature (HT) conditions with results of the statistical test with H_0_: log_2_(G/M) = 0 (a). Expression ratio of significantly shifting homeologs with results of the statistical test with H_0_: log_2_(G/M)_HT_ − log_2_(G/M)_CT_ = 0 (b) and log_2_(G/M)_HT_ − log_2_(G/M)_CT_ > 1.5.

To examine whether the broader transcriptional response in *murinum* was accompanied by changes in the relative contribution of its two subgenomes, we quantified homeolog expression bias between the G and M subgenomes under control and HT conditions. Across all analysed homeolog pairs, there was no evidence of a genome‐wide expression bias towards either subgenome under control conditions (*P* ~ 0.164; rank‐biserial *r* = −0.015) or under HT (*P* ~ 0.442; rank‐biserial *r* = −0.008; Figure 3a). Thus, neither control nor elevated temperature induced general dominance of one subgenome over the other in *murinum*.

Despite the absence of global bias, elevated temperature altered the relative expression of individual homeolog pairs. Quantifying the change in bias directly (Δ expression bias = log_2_(G/M)_HT − log_2_(G/M)_CT) revealed 702 of 11 106 homeologs with significant temperature‐dependent shifts towards one subgenome (padj < 0.01; |Δ expression bias| > 1.5; Figure 3b; Table S6). These shifts were almost evenly distributed between subgenomes, with 355 homeologs shifting towards G and 347 towards M. Only 151 of these 702 homeologs (~21.5%) overlapped with the DEGs, including 95 (26.8%) of those shifting towards G and 56 (16.1%) of those shifting towards M. This limited overlap indicates that temperature‐dependent homeolog bias represents an additional layer of transcriptomic response, rather than simply mirroring the genes identified as differentially expressed.

GO enrichment analysis of the 702 shifting homeologs further showed that the two subgenomes were associated with partly different functional tendencies under elevated temperature (Table 1). Homeologs shifting towards the G subgenome were enriched for stress‐ and environment‐associated functions, including ‘defence response’ (GO:0006952), ‘superoxide metabolic process’ (GO:0006801), and ‘methionyl‐tRNA aminoacylation’ (GO:0006431). The enrichment of superoxide metabolic process is consistent with the oxidative stress signature already detected among the broader heat‐responsive DEGs. ‘Defence response’ is likewise commonly induced under abiotic stress (Panahi, 2024; Yu et al., 2020). By contrast, homeologs shifting towards the M subgenome were enriched for functions related to core metabolism, RNA metabolism and translation, intracellular signalling, and reproductive cell division, including ‘lysine biosynthetic process via diaminopimelate’ (GO:0009089), ‘pseudouridine synthesis ‘(GO:0001522), ‘prolyl‐tRNA aminoacylation’ (GO:0006433), ‘tyrosine metabolic process' (GO:0006570), ‘reciprocal meiotic recombination’ (GO:0007131), ‘Rho protein signal transduction’ (GO:0007266), and ‘cytidine deamination’ (GO:0009972). Taken together, these enrichments suggest that under elevated temperature the G subgenome contributed more strongly to stress‐associated functions, whereas the M subgenome contributed more strongly to housekeeping, metabolic, and developmental processes.

**Table 1:** Significantly enriched GO terms among homeologs that showed significant expression shifts in response to high ambient temperature conditions

Subgenome	GO term	Description	Annotated homeologs	Observed shifting homeologs	Expected shifting homeologs	*P*‐value
G	GO:0006431	Methionyl‐tRNA aminoacylation	1	1	0.04	0.036
G	GO:0006952	Defence response	9	2	0.32	0.039
G	GO:0006801	Superoxide metabolic process	10	2	0.36	0.048
M	GO:0009089	Lysine biosynthetic process via diaminopimelate	6	2	0.22	0.02
M	GO:0001522	Pseudouridine synthesis	19	3	0.69	0.03
M	GO:0006433	Prolyl‐tRNA aminoacylation	1	1	0.04	0.04
M	GO:0006570	Tyrosine metabolic process	1	1	0.04	0.04
M	GO:0007131	Reciprocal meiotic recombination	1	1	0.04	0.04
M	GO:0007266	Rho protein signal transduction	1	1	0.04	0.04
M	GO:0009972	Cytidine deamination	1	1	0.04	0.04

To assess whether temperature responses in murinum also involved coordinated regulation of both homeologs without rebalancing their relative contribution, we additionally tested summed expression at the orthogroup level. This identified 1279 differentially expressed orthogroups out of 13 431 tested, whereas only 85 of these also showed a significant shift in homeolog expression bias. Thus, most responsive orthogroups changed in overall expression without a detectable change in the G:M ratio, consistent with coordinated regulation of both homeologs, suggesting that temperature‐dependent *cis*‐regulatory variation is largely conserved across the two subgenomes.

These results refine the expression pattern described above. In murinum, elevated temperature did not induce genome‐wide dominance of either subgenome under either condition, but instead altered expression through two more specific modes. Most responsive orthogroups changed in summed expression without a detectable change in the G:M ratio, consistent with coordinated regulation of both homeologs. Additionally, a subset of orthogroups showed significant shifts in homeolog expression ratios, indicating additional subgenome‐specific reweighting. An absence of genome‐wide subgenome dominance together with clear homeolog‐specific shifts has been reported in other allotetraploids (Akama et al., 2014; Akhunova et al., 2010; Takahagi et al., 2018; Wang et al., 2025). Such locus‐specific divergence may arise from ancestral regulatory differences, post‐polyploidisation divergence, subfunctionalisation, or a combination of both (Chaudhary et al., 2009; Hovav et al., 2008; Steige & Slotte, 2016). Similarly, coordinated regulation of homeologs, whether independently by each subgenome or in trans across subgenomes, has been suggested to contribute to post‐hybridisation evolution and trait variation (He et al., 2022; Shi et al., 2012). Together, coordinated homeolog regulation and homeolog‐specific ratio shifts may provide allopolyploids with additional regulatory flexibility that could potentially contribute to adaptability in ways not available to diploids.

### Distinct phenotypes but similar responses to elevated temperature

To compare phenotypic responses to elevated temperature in the material analysed here, we measured morphological traits (trichome density, plant height), growth‐related traits (SLA, vegetative biomass, spike extrusion) and reproductive traits (flowering time, spike number, reproductive biomass, harvest index) in the same 15 *glaucum* and 15 *murinum* accessions used throughout this study. This allowed us to ask whether the two subspecies differ in their trait values, whether elevated temperature affects them differently between the two subspecies, and whether these differences are reflected in trait covariation and overall plasticity.

**Figure 4:**
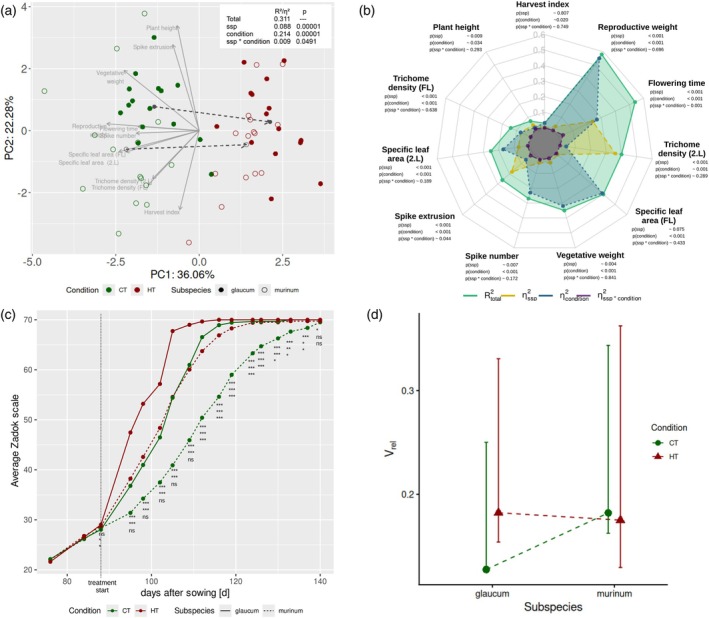
Multivariate and trait‐specific responses of *Hordeum murinum* subspecies to elevated temperature. (a) Principal components analysis (PCA) of phenotypic traits across the two subspecies and two temperature conditions (control and high temperature). Colours denote conditions, dot shapes denote subspecies. Grey arrows represent loadings of individual traits. Black dots indicate the subspecies × condition centre and the corresponding arrows depict how these centres shift in response to elevated temperature conditions. Inset: PERMANOVA summary indicating variance explained by subspecies, condition, and their interaction. (b) Spider plot showing variance explained (*R* ^2^ or η^2^) for each trait by subspecies, condition, and their interaction based on linear models. *P*‐values are indicated next to trait labels. (c) Mean developmental stage (Zadoks scale) over time for both subspecies under each temperature condition. Annotations denote significance levels at each timepoint. Effects from top to bottom: subspecies, condition, subspecies × condition (ns: not significant, *: *P* < 0.1, **: *P* < 0.05, ***: *P* < 0.01). (d) Trait integration strength (V_rel_) in *glaucum* and *murinum* under control (CT) and elevated temperature (HT) conditions. Points show observed values and error bars indicate 95% bootstrap confidence intervals; dashed lines connect CT and HT within subspecies.

Phenotypic measurements showed clear differences between *glaucum* and *murinum*. In multivariate trait space, subspecies was significant (*P* = 0.00001) and accounted for 8.7% of the total phenotypic variation (PERMANOVA; Figure 4a). Most individual traits, except for harvest index, SLA of the flag leaf, fertility, and germination rate, showed a significant subspecies effect (Figure 4b; Figure S5). The clearest difference between subspecies was recorded for flowering time (η^2^ = 0.24), with *glaucum* flowering on average 9 days earlier than *murinum* (109 versus 118 days), and this delay in development of *murinum* was already visible shortly after transfer from vernalisation into the growth chambers (Figure 4c). By contrast, differences in height, reproductive and vegetative biomass were statistically significant but small, unlike reports from some other polyploid systems in which tetraploids showed substantially larger organs or increased biomass production (Corneillie et al., 2019; Cseri et al., 2020; Sattler et al., 2016). Thus, the two subspecies differed clearly in phenotype, but these differences were driven more by development than by a general increase in plant size or biomass in the allotetraploid.

In comparison with these subspecies' differences, elevated temperature was the stronger source of phenotypic variation. In multivariate space, temperature explained nearly twice as much variation as subspecies (PERMANOVA *R* ^2^ = 0.214; Figure 4a), and all measured traits showed a significant condition effect with mostly larger effect sizes than the subspecies factor (Figure 4b; Figure S5). Biomass‐related traits were particularly sensitive, with reproductive biomass showing the strongest response, with a reduction of 47% in *murinum* and 42% in *glaucum*. Consistent with this, fertility, estimated as the proportion of filled spikelets, was significantly reduced under elevated temperature, although the effect size was small (Figure S5). Because reproductive biomass was measured from spikelets, reduced spikelet filling represents a component response that contributed to the decline in reproductive biomass under elevated temperature. Elevated temperature also accelerated development in both subspecies (η^2^ = 0.27), advancing flowering by about 6 days in *glaucum* and 12 days in *murinum* (Figure 4c). This pattern is consistent with previous work showing that reproductive processes are especially heat‐sensitive in cereals and in *H. murinum*, and that warming can shorten the period available for vegetative growth and biomass accumulation (Chano et al., 2021; Ejaz & von Korff, 2017; Farooq et al., 2011; Lan et al., 2025; Mishra et al., 2021; Prasad & Djanaguiraman, 2014). The decrease in SLA further suggests a shift towards thicker, smaller leaves under heat, a response often associated with drought‐related stress adjustment in grasses (Wellstein et al., 2017).

Despite these strong temperature effects, there was little evidence that the two subspecies responded differently to elevated temperature. In multivariate space, the subspecies × condition interaction was only marginally significant and had a very small effect size (PERMANOVA *P* ≈ 0.049, *R* ^2^ = 0.00869; Figure 4a). At the level of individual traits, flowering time was the only trait with a clearly significant interaction, while spike extrusion was just below the conventional 0.05 threshold, and both effect sizes were negligible (η^2^ = 0.03 and 0.02, respectively). Likewise, flowering time was the only trait where the two subspecies differed significantly (Figure S7). The multivariate plasticity index (nPCdpi) showed no subspecies effect (χ^2^ ≈ 0.073, *P* = 0.788), even though *glaucum* tended to show slightly greater variation among genotypes (Figure S6). Thus, both subspecies exhibited strong phenotypic responses to elevated temperature, but did so in similar ways. This similarity was also reflected in trait covariation. Trait correlation matrices were broadly similar across subspecies and temperature treatments (Figure S8). In all four subspecies × condition groups (i.e. *glaucum* CT, *glaucum* HT, *murinum* CT, *murinum* HT), plant height was positively and significantly (*P* ≤ 0.01) correlated with spike extrusion, and trichome densities of the flag leaf and second leaf were likewise positively associated. Some individual correlations varied in strength across groups, particularly those involving flowering time and biomass‐related traits, but no clear reorganisation of the overall correlation structure was apparent. This impression was supported by the analysis of trait integration strength, quantified as the mean squared off‐diagonal correlation (V_rel_; Pavlicev et al., 2009). Across the two conditions, mean V_rel_ was 0.128 under control and 0.191 under elevated temperature in *glaucum*, compared with 0.182 and 0.175, respectively, in *murinum*. (Figure 4d; Figure S8). However, bootstrap confidence intervals for the effects of subspecies (0.024, 95% CI: −0.067 to 0.123), condition (0.024, 95% CI: −0.068 to 0.119), and their interaction (−0.062, 95% CI: −0.250 to 0.119) all overlapped with zero. Thus, these results indicate that elevated temperature altered trait values in both subspecies without substantially changing the overall strength or structure of trait integration, and that *glaucum* and *murinum* did not differ clearly in how trait covariation responded to elevated temperature. Similar results have been reported in other polyploid comparisons, where environmental treatments strongly affect phenotype, but differences between cytotypes in plasticity or performance remain small or inconsistent (Kornstad et al., 2022; Tossi et al., 2022). By contrast, some allopolyploids do show greater phenotypic homeostasis across thermal regimes, as reported for *Coffea arabica* L., suggesting that such effects may be lineage‐ and stress‐specific rather than a general consequence of genome duplication (Bertrand et al., 2015).

Taken together, these results show that *glaucum* and *murinum* differ in morphological and developmental traits, especially in phenology, but respond similarly to elevated temperature. If reproductive biomass is taken as a proxy for performance under elevated temperature, *murinum* did not outperform *glaucum*, and we likewise found no evidence of greater plasticity in the allotetraploid. This contrasts with the common expectation that polyploids are generally more stress tolerant or more plastic due to theories of gene redundancy and epigenetic buffering (Comai, 2005; Li et al., 2024; Mousavizadeh et al., 2022; Parisod & Broennimann, 2016; Ramsey, 2011), and instead supports a more nuanced view in which any advantage of polyploidy depends on lineage history, the nature of the stressor, and the timescale considered (Li et al., 2024; Soltis et al., 2014; Tossi et al., 2022). In the context of our overall comparison, the striking point is that these largely shared phenotypic responses to elevated temperature occurred despite the broader and more strongly partitioned transcriptomic response observed in *murinum*, and that this did not translate into greater fitness stability.

## CONCLUSION

Our study asked whether allopolyploidy translates into greater reproductive stability under changing environmental conditions, whether such an advantage is associated with altered responses of morphological and developmental traits, and whether it is accompanied by a more extensive transcriptional response.

In *Hordeum murinum*, we found no evidence that allopolyploidy conferred greater reproductive stability under the temperature regime tested here. *Murinum* showed neither greater plasticity nor better performance than *glaucum*. The clearest differences instead emerged at the molecular level: *glaucum* harboured higher gene‐level nucleotide diversity and *murinum* showed a broader transcriptional response to elevated temperature. Within *murinum*, that response involved substantial subgenome‐specific contributions and temperature‐dependent shifts in homeolog expression bias.

More generally, our results suggest that phenotypic differences between diploid and allotetraploid subspecies and divergent transcriptional responses to elevated temperature do not necessarily translate into different fitness outcomes. Rather than conferring reproductive superiority, the broader transcriptional response of *murinum* may represent an alternative route to comparable performance under temperature variation.

## MATERIAL AND METHODS

### Plant material and cultivation

We assembled a collection of 253 accessions of *murinum* and 63 accessions of *glaucum* described in Villhauer et al. (2026) and complemented by genebank accessions (Table S1). Bioclimatic variables were extracted from WorldClim (Fick & Hijmans, 2017), and 15 accessions per subspecies were selected to evenly represent the PCA space of these climatic variables. The experiment was conducted as a completely randomised design, with three replicates per accession, resulting in a total of 90 plants per experimental treatment: control and high ambient temperature. Seeds were sown at a depth of 2 cm in 96‐well trays filled with a freshly mixed substrate consisting of 99.6% (v/v) Mini tray soil (MIM800, Balster Einheitserdewerk, Frondenberg, Germany) and 0.4% (v/v) Osmocote Exact Standard 3–4 M (Scotts Company LLC, Ohio, USA). Seedlings were initially grown for 8 days under long‐day conditions (16 h light/8 h dark) at a control temperature regime (20°C day/16°C night). After germination, plants underwent vernalisation for 68 days at 6°C under short‐day conditions (8 h light/16 h dark). Once vernalisation was complete, seedlings were transplanted into 9 × 9 cm pots containing a mixture of 93% (v/v) Einheitserde ‘ED73’ peat soil (Einheitserde Werkverband e.V., Sinntal Altengronau, Germany), 6.6% (v/v) sand, and 0.4% (v/v) Osmocote Exact Standard 3–4 M. Plants were allowed to acclimate for 7 days under long‐day, control temperature conditions. Following acclimation, one set of plants remained under control conditions (CT‐LD), while a second set was moved to a high‐temperature (HT) regime (28°C day/24°C night) under the same photoperiod.

### RNA extraction and sequencing

Ten days after transferring plants to HT conditions, we collected tissue from the middle section of the second leaf from all accessions in CT and HT. Because the experimental unit was the subspecies, we sampled only one replicate per condition, resulting in 15 replicates for each of the two subspecies × condition combinations. Total RNA was extracted using the RNeasy kit (Qiagen, Hilden, Germany). Poly(A)‐enriched mRNA library preparation was performed by Novogene Europe, who subsequently sequenced the libraries using 150 bp paired‐end Illumina reads. *Hordeum murinum* samples were sequenced to a depth of 10 Gb per sample, whereas *glaucum* samples were sequenced to 5 Gb per sample.

### Gene diversity and divergence across genomes

Raw reads of *glaucum* and *murinum* samples were mapped against the reference genomes of BCC2017 (*glaucum*) and BCC2009 (*murinum*; Mascher et al., 2025), respectively, using ‘STAR’ (v2.7.10a; Dobin et al., 2013). Only uniquely mapped reads were retained, and gene expression was quantified simultaneously with the – quantMode GeneCounts option. To investigate patterns of nucleotide diversity and divergence among subgenomes, RNA‐seq reads from *murinum* were separated by subgenome from the BAM files and exported in FASTQ format. This resulted in one FASTQ file per *glaucum* accession and two per *murinum* accession corresponding to reads assigned to the G and M subgenomes. These separated reads were then mapped to the *glaucum* reference genome BCC2017 (Mascher et al., 2025) using STAR (v2.7.10a; Dobin et al., 2013). Only uniquely mapped reads were retained. Variant calling was performed using bcftools (v1.22; Danecek et al., 2021), applying the following filtering criteria: ‐‐types snps, ‐‐min‐alleles 2, ‐‐max‐alleles 2, −e ‘GT==“het” & DP < 30’. Further filter criteria applied in VCFtools (v0.1.16; Danecek et al., 2011) were ‐‐minDP 5, ‐‐minQ 30, ‐‐minGQ 30 and ‐‐max‐missing 0.8. For each gene, nucleotide diversity (π) was calculated within each group, and pairwise measures of allelic differentiation (Jost's D) calculated between groups using custom R functions (R Core Team, 2025).

### Differential expression analysis

For gene expression analyses, we used the expression quantification from the mapping procedure described above. Initial preprocessing of raw reads included filtering of lowly expressed genes, defined as those not exceeding 1 count per million (CPM) in at least two samples, and the removal of outlier accessions based on principal component analysis (PCA). No outliers were detected in *glaucum*, yet two accessions (IG168058 and IG168079) remained to be outlying groups relative to the other *murinum* HT accessions even after correcting for technical variation in read depth and composition with DESeq2's size‐factor normalisation (Love et al., 2014). They were excluded in both CT and HT to avoid technical biases, leaving 13 *murinum* accessions for the analyses. Normalisation of raw counts was performed using the DESeq2 median‐of‐ratios method to account for differences in sequencing depth and library composition. Differential expression was assessed using DESeq2, modelling individual accession identity as a covariate to account for paired samples. Wald tests were performed for each gene, and log_2_ fold‐change shrinkage was applied using the apeglm method to obtain stable effect size estimates (Zhu et al., 2019). Genes with an adjusted *P*‐value (FDR) <0.01 and absolute log_2_ fold change >1 were considered significantly differentially expressed. This analysis was performed separately for *glaucum* and *murinum*.

Functional enrichment of differentially expressed genes was performed for the ‘Biological Process’ ontology using topGO (Alexa & Rahnenführer, 2025). GO annotations were extracted from a subspecies‐specific feature table from Mascher et al. (2025), and enrichment was assessed relative to the complete set of expressed genes. The weight01 algorithm was used to identify significantly overrepresented terms (*P* < 0.05).

### Quantification of subgenome bias and shift

Using previously published homeolog relationships between the two *murinum* subgenomes (Hellwig et al., 2025), we organised raw transcript counts from the 15 *murinum* accessions into a matrix in which each homeolog was represented by counts from all samples and both subgenomes. For each homeologous gene pair, this matrix contained one count for the G and one for the M subgenome of *murinum* under both control temperature (CT) and high‐temperature (HT) conditions.

We retained homeologous gene pairs with counts greater than 1 count per million in at least two samples (edgeR v4.6.3; Chen et al., 2025). Library size normalisation factors were computed on the combined G + M count matrix using the ‘trimmed mean of M values’ (TMM) method (edgeR::calcNormFactors). These TMM‐derived normalisation factors were converted to sample‐relative scaling factors and applied back to the original G and M columns, ensuring that subgenome‐specific counts were scaled consistently using the same sample‐level library factors. All downstream expression analyses were performed on these scaled counts.

Scaled G and M counts were averaged across the 13 accessions within each condition (CT and HT). Per‐homeolog subgenome expression bias was then calculated as the log_2_ ratio, log_2_(G/M), for each condition. Overall subgenome bias within each condition was assessed using Wilcoxon signed‐rank tests on the distribution of log_2_(G/M) values across genes, with effect size quantified using rank‐biserial correlation (effectsize v1.0.1; Ben‐Shachar et al., 2020).

To identify genes exhibiting temperature‐dependent shifts in subgenome expression bias, we calculated per‐homeolog paired differences in bias (Δbias = log_2_(G/M)_HT_ − log_2_(G/M)_CT_) using matched observations across conditions. Paired Wilcoxon signed‐rank tests were applied per homeolog, and resulting *P*‐values were adjusted for multiple testing using the Benjamini & Hochberg procedure (Benjamini & Hochberg, 1995).

### Phenotyping and statistical trait analyses

After repotting, plant development was monitored twice weekly until the grain‐filling stage (stage 70), guided by the Zadoks scale (Zadoks et al., 1974). At ripening, plants were harvested by cutting just above the soil. The flag leaf and the second youngest leaf beneath it were measured for area, then dried and weighed to determine biomass and calculate specific leaf area (SLA). Remaining plant material was dried, with seeds and vegetative tissue weighed separately. Reproductive traits were assessed at harvest by counting reproductive tillers, measuring the length of the longest tiller, and recording spike extrusion relative to the auricle. Spikes that ripened without extending past the auricle were assigned negative values for spike extrusion. Fertility was assessed for the first replicate of plants, plus a random subset of the second and third replicate, by determining the proportion of 50 randomly selected spikelets that had developed a seed. Germination rate of the harvested seeds was assessed by testing the germination of 20 seeds per accession on Petri dishes within 7 days. Anther extrusion was evaluated visually, with plants considered positive if approximately 30% or more of the main spike displayed extruded anthers.

Statistical analyses of the phenotyped traits were conducted in the R environment (R Core Team, 2025). Phenotypic traits were analysed using linear models (type II sum of squares) to assess the effects of subspecies, growth condition, and their interaction. The explained variation of the total model (*R* ^2^) and the individual factors (η^2^) were calculated using the R package ‘effectsize’ (Ben‐Shachar et al., 2020).

Permutational multivariate analysis of variance (PERMANOVA) was conducted with 99 999 permutations to estimate *R* ^2^ and η^2^ of the multivariate trait space using the R package ‘vegan’ (Jari, 2018).

To assess whether relationships among traits were retained across subspecies and temperature treatments, we calculated pairwise Pearson correlations among phenotypic traits for each combination of subspecies × condition (*glaucum* CT, *glaucum* HT, *murinum* CT, *murinum* HT) using genotype means. Harvest index was excluded because it is a derived trait and could therefore introduce non‐independence into the correlation structure. Correlation matrices were calculated from the selected traits and visualised separately for each subspecies × condition combination. To summarise overall trait integration, we calculated V_rel_ as the mean squared off‐diagonal correlation for each subspecies × condition combination. For correlation matrices, this metric is equivalent to the relative eigenvalue variance and provides a measure of the overall strength of covariation among traits (Pavlicev et al., 2009). From the four group‐specific values, we derived contrasts representing the effects of subspecies, condition, and their interaction. Uncertainty was estimated by stratified bootstrap resampling within each subspecies × condition combination, recalculating V_rel_ and all contrasts for each bootstrap sample. We report observed estimates together with 95% bootstrap confidence intervals.

To quantify the multivariate plastic response of each genotype to HT conditions, we calculated a statistic conceptually similar to the relative distance plasticity index introduced by Valladares et al. (2006). Throughout this manuscript, we refer to this statistic as nPCdpi, which serves as a multivariate indicator of phenotypic plasticity in response to environmental variation. We performed a principal component analysis (PCA) on the trait dataset without averaging over biological replicates. After inspecting the scree plot, we extracted the top six principal components (PCs) and weighted eigenvalues according to the proportion of variance each PC explained. This weighting ensures that PCs contributing more to overall variation had greater influence on the distance calculation. Pairwise Euclidean distances between all replicate combinations were computed within each genotype across treatments. Only distances between replicates under different conditions were considered, ensuring that the metric captured the separation attributable to the treatment. The median of these distances was then taken as a summary statistic representing the genotype‐specific response to the treatment in the phenotypic PCA space. Due to the non‐normal distribution of this statistic, we tested for a subspecies effect using a Kruskal–Wallis test.

## AUTHOR CONTRIBUTIONS

TH: Conceptualisation, investigation, methodology, software, formal analysis, visualisation, writing – original draft, data curation, project administration. MMT: Investigation. HV: Resources, writing – review and editing. AB: Resources, funding acquisition, writing – review and editing. MvK: Resources, funding acquisition, writing – review and editing, supervision.

## CONFLICT OF INTEREST

The authors declare that they have no competing interests.

## Supporting information


**Figure S1.** Differential expression results of subspecies glaucum grown in control and elevated temperature conditions. (A) Counts of DEGs. (B) Volcano plot. (C) Heatmap of the 100 genes with the lowest adjusted *P*‐values. Colours in the heatmap indicate log2 expression.
**Figure S2.** Differential expression results of subspecies murinum grown in control and elevated temperature conditions. (A) Counts of DEGs. (B) Volcano plot. (C) Heatmap of the 100 genes with the lowest adjusted *P*‐values. Colours in the heatmap indicate log2 expression.
**Figure S3.** GO term network of differentially expressed genes of *glaucum* grown in control and elevated temperature conditions. GO terms are coloured according to *P*‐values from yellow (high) to red (low).
**Figure S4.** GO term network of differentially expressed genes of *murinum* grown in control and elevated temperature conditions. GO terms are coloured according to *P*‐values from yellow (high) to red (low).
**Figure S5.** Violin plots of individual traits measured in control and elevated temperature conditions for *glaucum* and *murinum*.
**Figure S6.** Multivariate phenotypic plasticity index (nPCdpi) of *glaucum* and *murinum* under elevated temperature.
**Figure S7.** Relative distance plasticity index (RDPI) between control and elevated temperatures of measured traits in *glaucum* and *murinum*. (Fertility and germination rate were omitted here, since they were not measured for all replicates).
**Figure S8.** Pearson's correlation of phenotypic traits for each subspecies × condition combination. Black and grey values indicate significant correlation with *P* ≤ 0.01.

**Table S1.** Metadata of the included germplasm collection.
**Table S2.** Gene‐wise nucleotide diversity and Jost's D.
**Table S3.** Gene‐wise differential expression results of subspecies *glaucum* grown in control and elevated temperature conditions.
**Table S4.** Gene‐wise differential expression results of subspecies *murinum* grown in control and elevated temperature conditions.
**Table S5.** Significant gene ontology terms of *glaucum* and *murinum*.
**Table S6.** Subspecies' average expression of homeologs, log2 fold change and homeolog expression bias.
**Table S7.** Orthogroup‐wise differential expression results of subspecies murinum grown in control and elevated temperature conditions. G genome gene IDs an protein annotations were used as representative of the orthogroup.
**Table S8.** Phenotypic trait measurements.

## Data Availability

Raw data, a selection of intermediate results and scripts were stored in our FAIR data publication which can be found under DOI: https://doi.org/10.60534/ykap9‐gjj27.
