# Supplementary material for: Diploid‐ and tetraploid wall barley exhibit alternative molecular responses but comparable reproductive performance in response to different ambient temperature regimes
Source: Plant J. 2026 Jul 25;127(2):e71051. doi: 10.1111/tpj.71051 (PMC13401425; doi:10.1111/tpj.71051)
Supplement: Supplementary file 1 — Figure S1. Differential expression results of subspecies glaucum grown in control and elevated temperature conditions. (A) Counts of DEGs. (B) Volcano plot. (C) Heatmap of the 100 genes with the lowest adjusted P‐values. Colours in the heatmap indicate log2 expression. Figure S2. Differential expression results of subspecies murinum grown in control and elevated temperature conditions. (A) Counts of DEGs. (B) Volcano plot. (C) Heatmap of the 100 genes with the lowest adjusted P‐values. Colours in the heatmap indicate log2 expression. Figure S3. GO term network of differentially expressed genes of glaucum grown in control and elevated temperature conditions. GO terms are coloured according to P‐values from yellow (high) to red (low). Figure S4. GO term network of differentially expressed genes of murinum grown in control and elevated temperature conditions. GO terms are coloured according to P‐values from yellow (high) to red (low). Figure S5. Violin plots of individual traits measured in control and elevated temperature conditions for glaucum and murinum. Figure S6. Multivariate phenotypic plasticity index (nPCdpi) of glaucum and murinum under elevated temperature. Figure S7. Relative distance plasticity index (RDPI) between control and elevated temperatures of measured traits in glaucum and murinum. (Fertility and germination rate were omitted here, since they were not measured for all replicates). Figure S8. Pearson's correlation of phenotypic traits for each subspecies × condition combination. Black and grey values indicate significant correlation with P ≤ 0.01. [file TPJ-127-0-s002.pdf]

A

Category	Count	Percent
Total DE genes	1740	7.94
Up−regulated in HT	1037	4.73
Down−regulated in HT	703	3.21

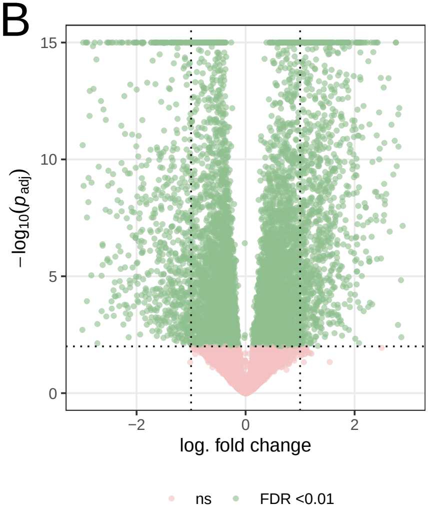

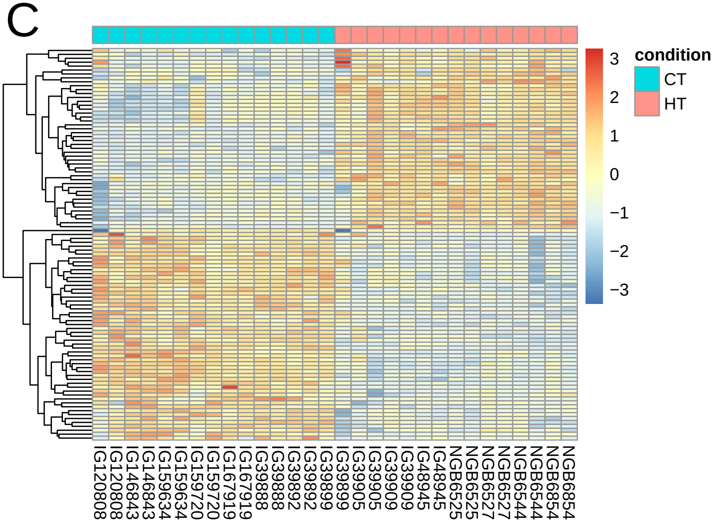

Figure S1: Differential expression results of subspecies *glaucum* grown in control and elevated temperature conditions. A: Counts of DEGs. B: Volcano plot. C: Heatmap of the 100 genes with the lowest adjusted p-values. Colours in the heatmap indicate $\log_2$ expression.

A

Category	Count	Percent
Total DE genes	3769	10.20
Up−regulated in HT	2594	7.02
Down−regulated in HT	1175	3.18

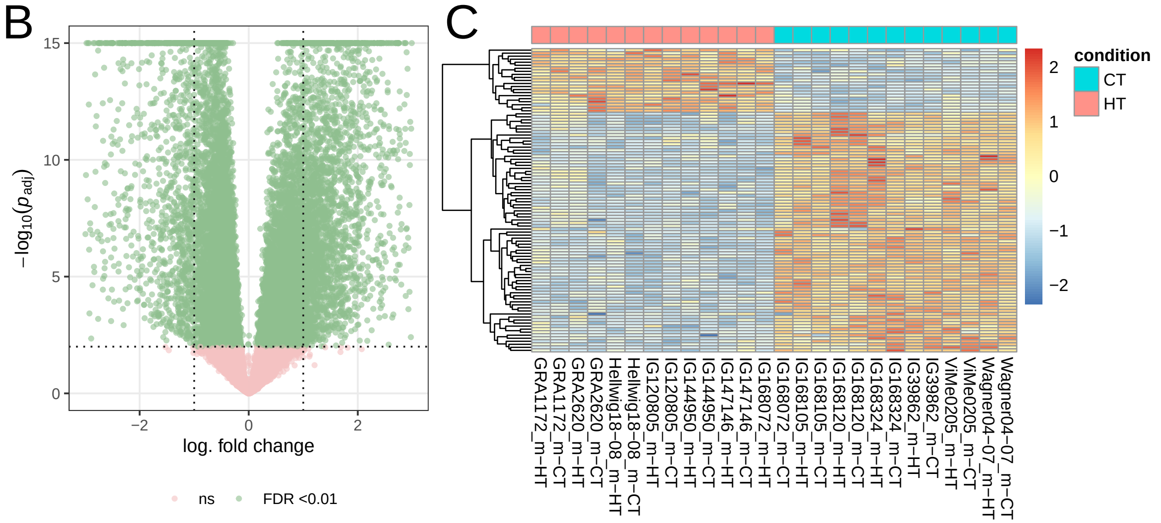

Figure S2: Differential expression results of subspecies *murinum* grown in control and elevated temperature conditions. A: Counts of DEGs. B: Volcano plot. C: Heatmap of the 100 genes with the lowest adjusted p-values. Colours in the heatmap indicate $\log_2$ expression.

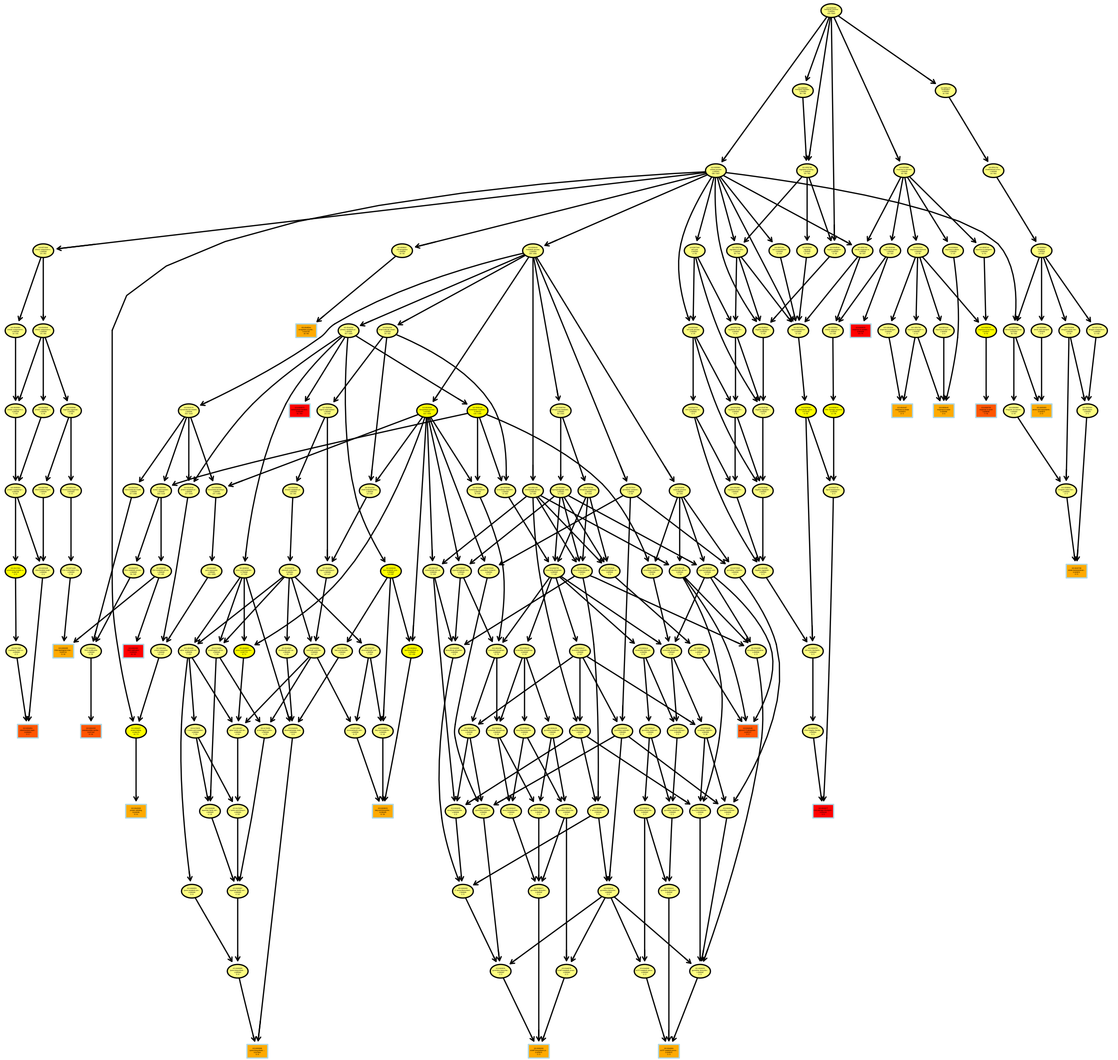

Figure S3: GO term network of differentially expressed genes of *glaucum* grown in control and elevated temperature conditions. GO-terms are coloured according to p-values from yellow (high) to red (low).

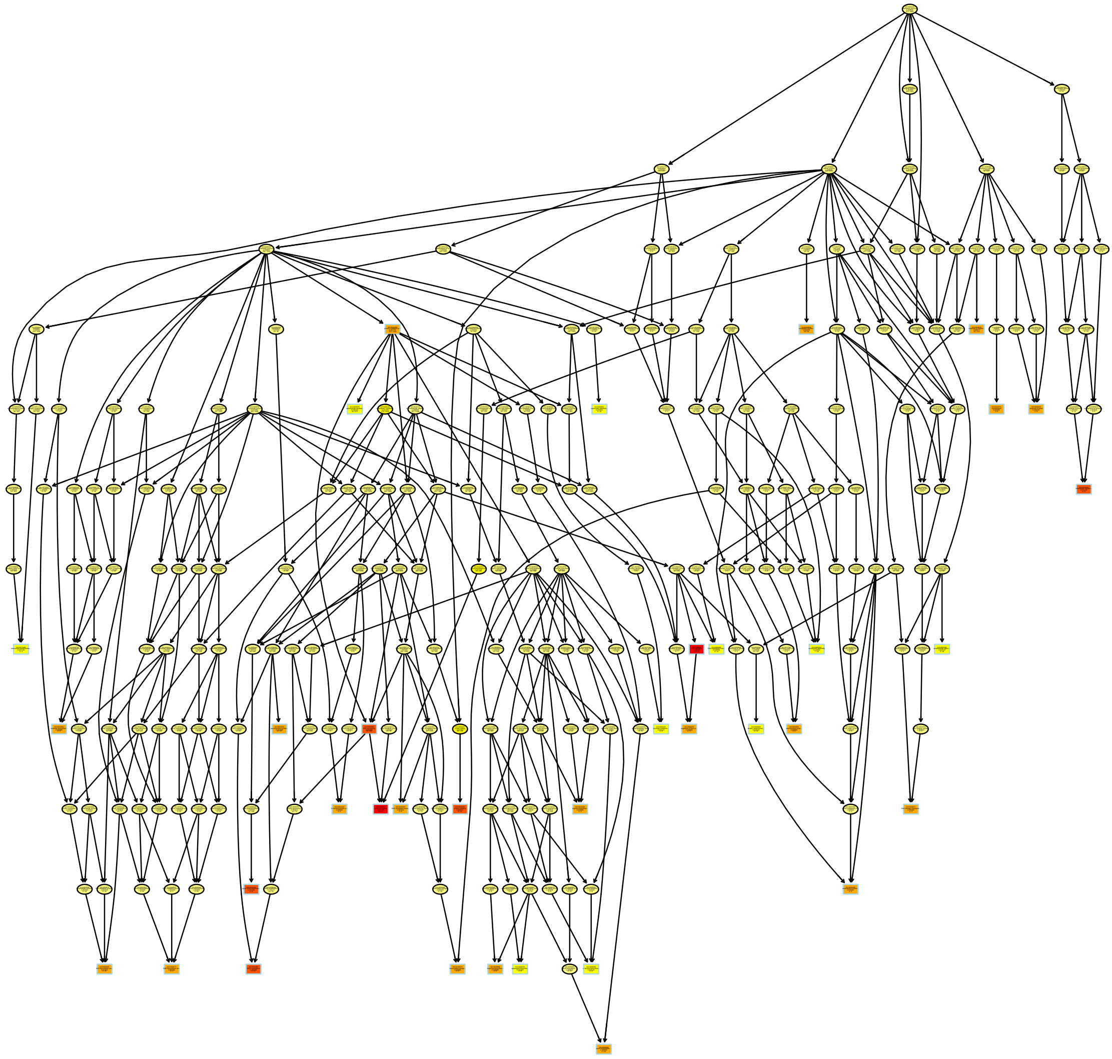

Figure S4: GO term network of differentially expressed genes of *murinum* grown in control and elevated temperature conditions. GO-terms are coloured according to p-values from yellow (high) to red (low).

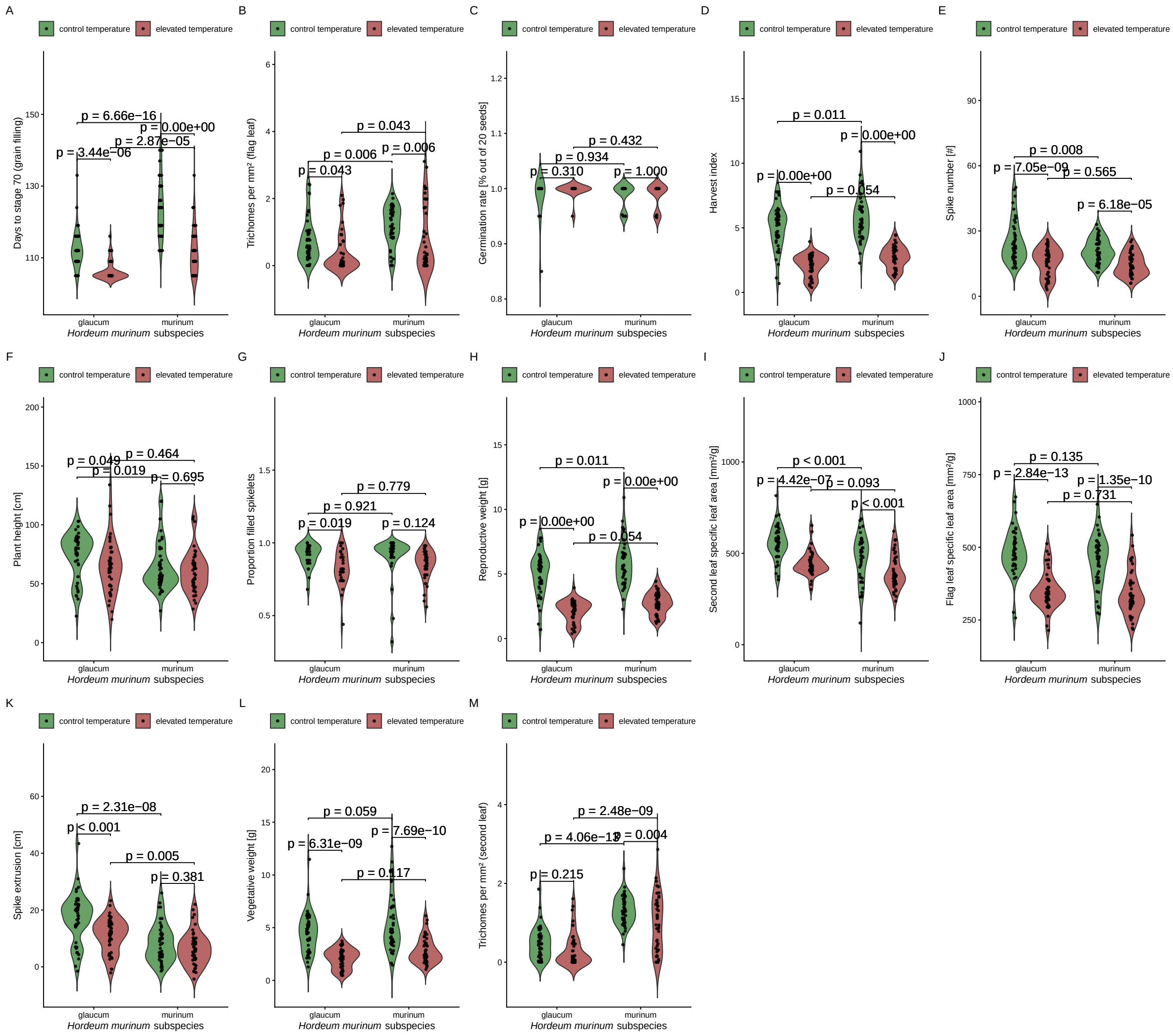

Figure S5: Violin plots of individual traits measured in control and elevated temperature conditions for *glaucum* and *murinum*.

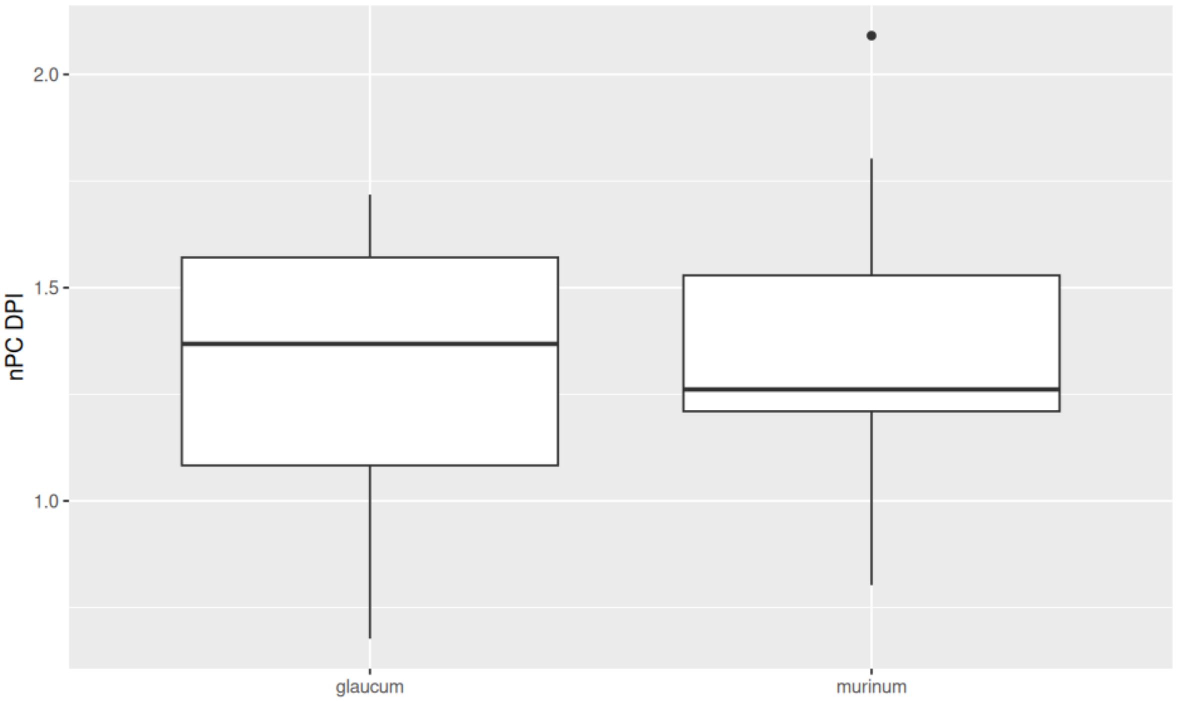

Figure S6: Multivariate phenotypic plasticity index (nPCdpi) of *glaucum* and *murinum* under elevated temperature.

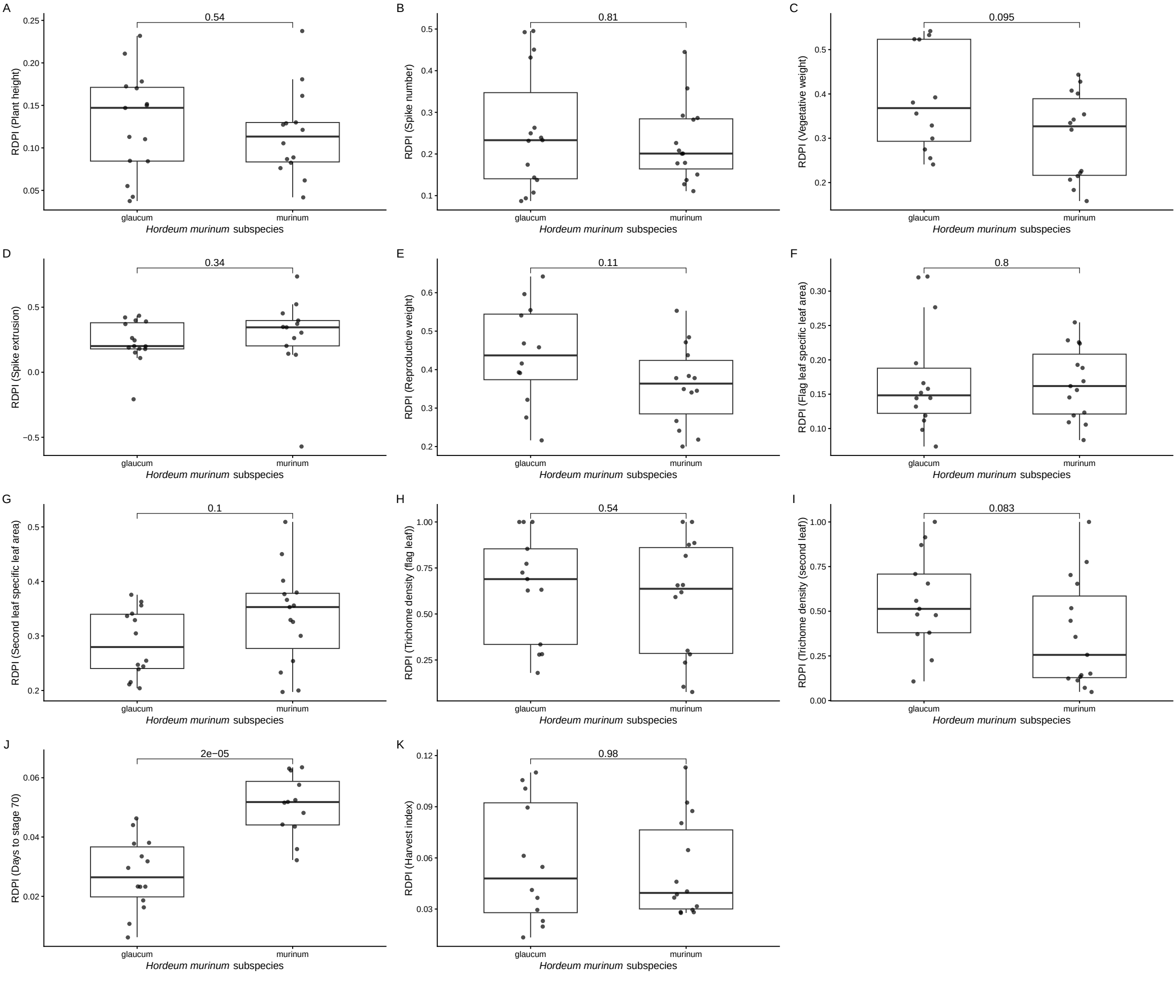

Figure S7: Relative Distance Plasticity Index (RDPI) between control and elevated temperatures of measured traits in *glaucum* and *murinum*. (Fertility and germination rate were omitted here, since they were not measured at all replicates)

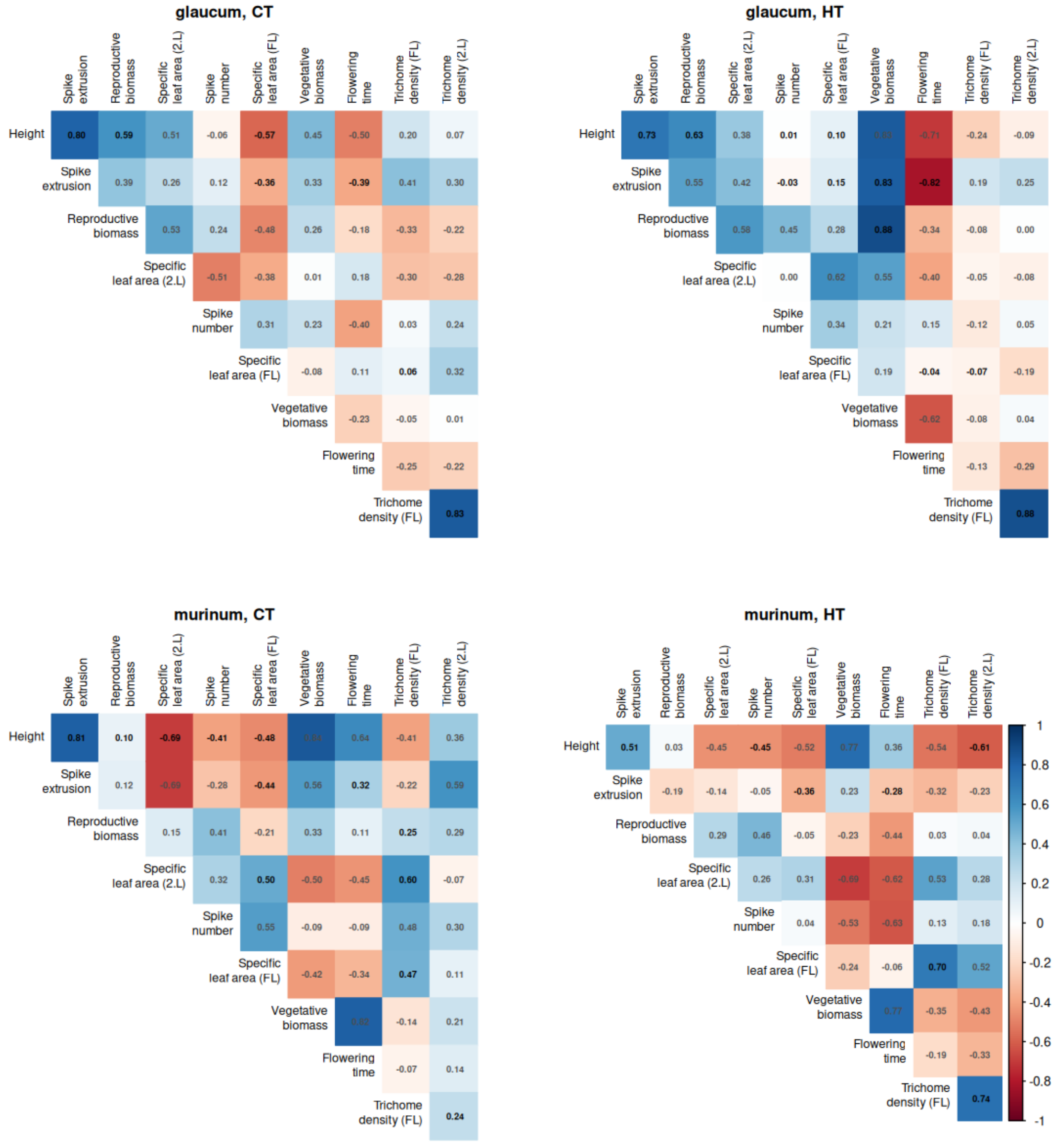

Figure S8: Pearson’s correlation of phenotypic traits for each subspecies x condition combination. Black and grey values indicate significant correlation with $p \leq 0.01$.
